# Herpes Zoster Ophthalmicus Secondary to Periorbital Cellulitis

**DOI:** 10.7759/cureus.60453

**Published:** 2024-05-16

**Authors:** Krishna Sheth, Cody Lee, Maha Jangda, Yaw Baah

**Affiliations:** 1 Internal Medicine, Garnet Health Medical Center, Middletown, USA; 2 Internal Medicine, Touro College of Osteopathic Medicine, Middletown, USA; 3 Medicine, Garnet Health Medical Center, Middletown, USA

**Keywords:** herpes zoster ophthalmicus, preseptal cellulitis, herpes zoster virus, varicella zoster virus, periorbital cellulitis

## Abstract

Varicella zoster virus (VZV) infection, also commonly known as chickenpox, is a communicable disease most often contracted in childhood via contact, airborne, or droplet transmission. After about a two-week incubation period, patients can experience a prodromal phase, which includes a pruritic vesicular blistering rash with associated constitutional symptoms such as fever, headache, malaise, muscle aches, fatigue, and sore throat. Symptoms are often self-limiting and only require supportive care and observation. We report a case of a 54-year-old female who presented with an unusual background history and was found to have a rare manifestation of herpes zoster virus, presenting as herpes zoster ophthalmicus (HZO).

## Introduction

The hallmark clinical signal of varicella zoster virus (VZV) is the exanthem phase defined by an intensely pruritic rash that contains lesions at various stages of development. The rash starts centrally on the trunk and eventually spreads to the extremities. Rarely do the lesions appear on the palms and soles. Characteristic stages of cutaneous lesions follow a maculopapular rash that evolves into vesicles filled with clear fluid, which can become tense and eventually rupture or crust to form a scab. After the primary infection when systemic and cutaneous symptoms subside, the virus enters a latent and dormant phase within sensory neurons. It can then later reemerge, often several years after primary VZV infection, as herpes zoster (HZ). This reemergence usually occurs during periods of stress or immunosuppression as a painful, vesicular rash that follows a dermatomal pattern [1]. Complications that can arise after the acute phase infection include secondary bacterial superinfection, scarring of cutaneous lesions, and HZ, otherwise known as shingles. HZ is the subsequent viral reactivation of a latent VZV infection. Although less contagious than primary VZV infection, standard health and safety protocol for transmission prevention is advised. Antiviral therapy is usually reserved for severe infections and immunocompromised patients [1].

Herpes zoster ophthalmicus (HZO) is a subvariant of HZ. Of the patients with HZ infection, only 10%-20% will present with HZO, and about 50% of HZO cases have true ocular disease. HZO is a relatively rare manifestation of HZ that can potentially lead to serious complications such as vision loss if not recognized and treated appropriately. HZO is a relatively uncommon form of shingles that affects the ophthalmic division (V1) of the trigeminal nerve [1]. It manifests as a severely painful maculopapular or vesicular rash in a unilateral dermatomal pattern with associated symptoms including fever, conjunctivitis, and intraocular infection [2]. Ocular inflammation is the most concerning feature of HZO. The nasociliary nerve, a branch of the ophthalmic division, is responsible for intraocular innervation. Therefore, uveitis, iritis, conjunctivitis, keratitis, and optic neuritis may be present [1,2]. VZV is highly contagious during this period, and precautions should be taken to prevent the spread, especially to high-risk individuals [3].

## Case presentation

A 54-year-old female with a notable history of VZV infection in childhood presented to the emergency department from home with right eye pain, swelling, and facial rash that began five days prior to arrival at the hospital. Her symptoms began soon after a piece of firewood flew and struck her right eye while she was chopping firewood in her backyard. The patient reactively wiped her eye with a gloved hand that was contaminated with chicken feces from her backyard coop. Shortly after the incident, the patient noticed mild swelling, pain in her right eye, and a few superficial abrasions. The following day, she reported increased swelling of the area and a painful, blistering facial rash associated with pinprick sensations over the lesions. In the emergency department, the patient stated the blisters began to open and had a clear discharge. She also noticed a firm, small mass behind her right ear. The patient did not receive prior herpes zoster immunizations. She also endorsed living in a heavily wooded area and prior Lyme disease infections.

**Figure 1:**
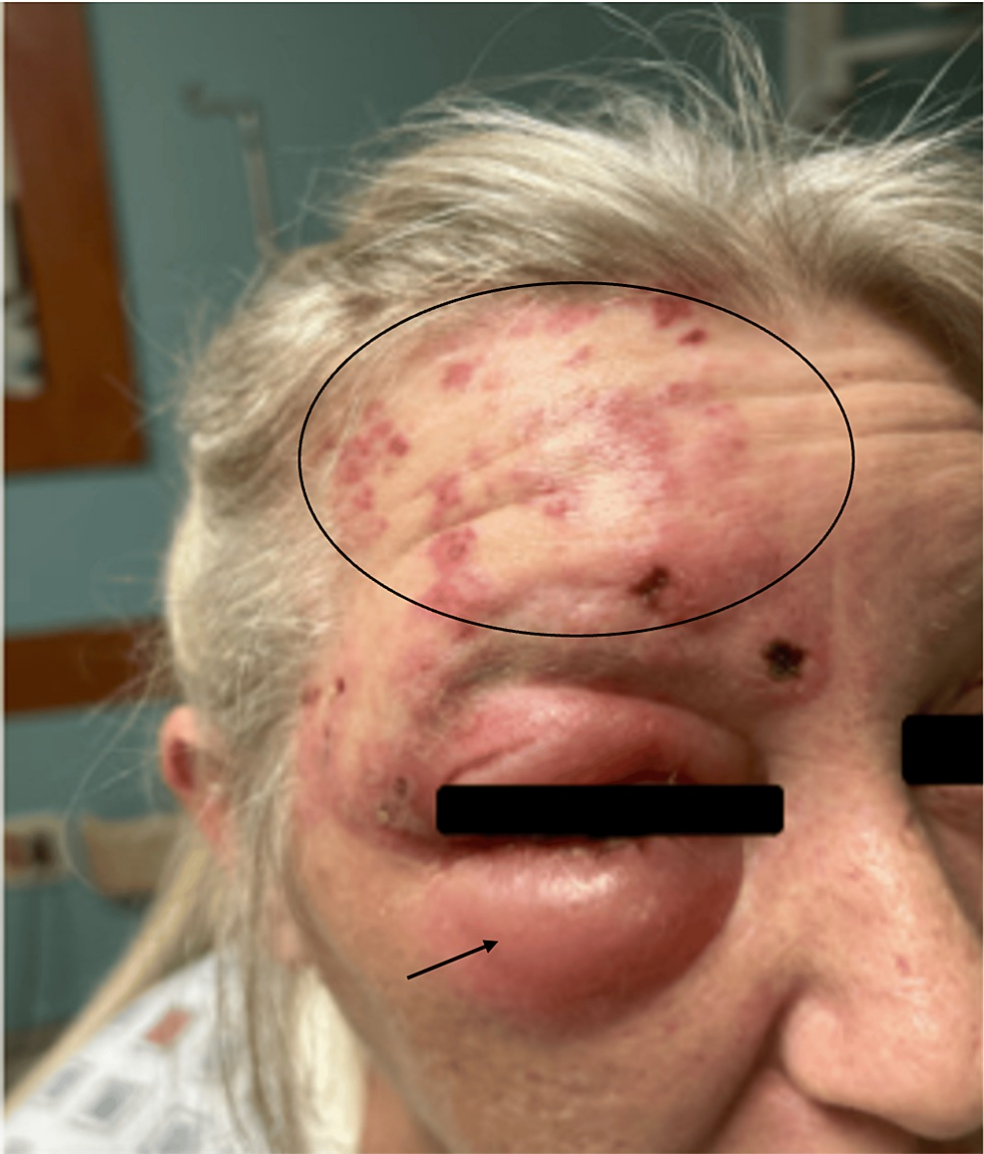
Vesicular rash (circle) affecting the ophthalmic division of the trigeminal nerve prior to treatment initiation and periorbital cellulitis (arrow)

On initial physical examination, periorbital edema, erythema, and tenderness were noted. Her skin had a vesicular rash in various stages of healing. The rash, as seen in Figure 1, appeared in a dermatomal pattern on the right forehead, extended back toward her scalp, did not cross the midline, and was exquisitely tender to light touch. The vesicular lesions also expressed a clear discharge. No other rashes or skin lesions were appreciated. There was no extraocular muscle weakness, facial droop, or other signs of cranial nerve deficits. The right conjunctiva was mildly injected; however, visual acuity was intact, and pupils were equal, round, and reactive to light. Further ophthalmologic survey showed no evidence of keratitis, uveitis, or inner eye vesicular lesions. A fluorescein examination performed at bedside did not reveal any foreign bodies, corneal abrasions, or ulcerations. Extraocular motion was full and intact. She was placed on contact isolation with droplet precautions based on high clinical suspicion of herpes zoster infection.

**Figure 2:**
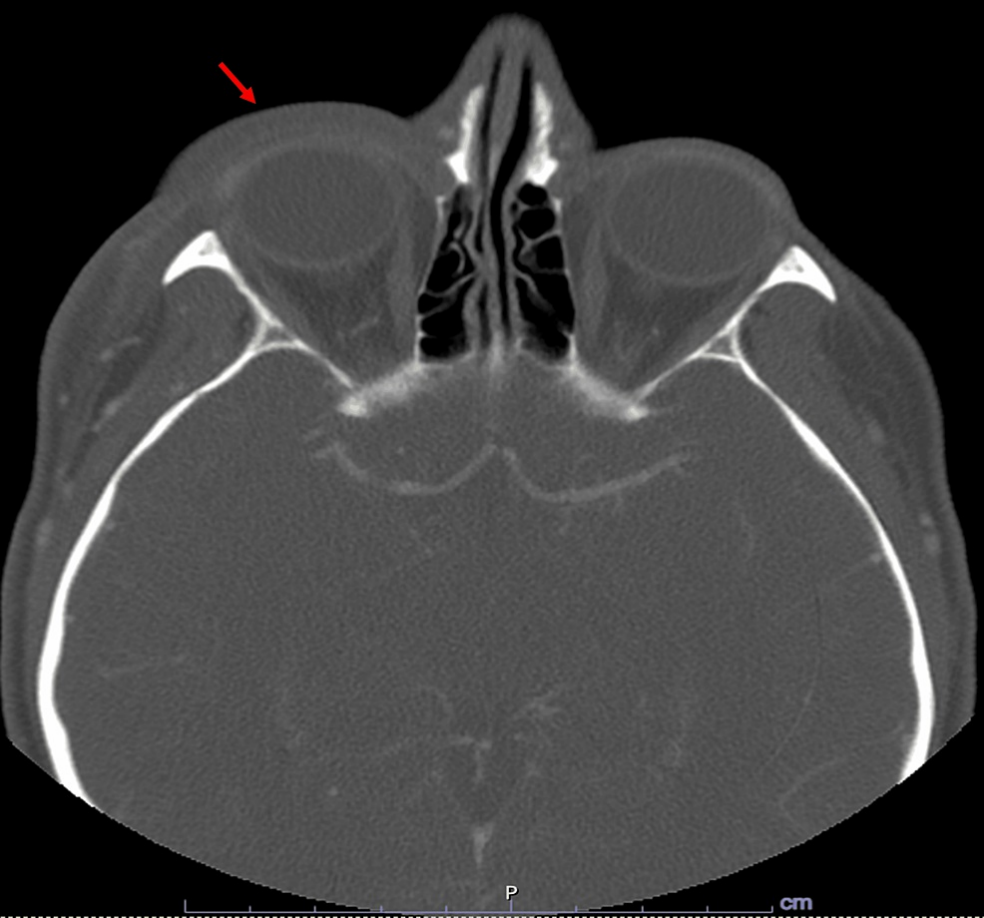
Axial contrast-enhanced CT of orbits and sella turcica showing right periorbital and preseptal soft tissue swelling (red arrow) CT: computed tomography

On admission, the patient was afebrile, had a normal oxygen saturation and elevated blood pressure at 148/90, and was tachycardic at 110 beats/minute. Complete blood count results were significant for polycythemia with a hemoglobin of 15. However, no evidence of leukocytosis or electrolyte abnormalities was noted. Subsequent blood and urine cultures were negative. Lyme antibodies and methicillin-resistant *Staphylococcus aureus* (MRSA)-SA polymerase chain reaction (PCR) were unremarkable. Computed tomography of orbits and sella turcica with contrast revealed right periorbital and preseptal soft tissue swelling with no evidence of subperiosteal abscess or retrobulbar collection, as seen in Figure 2.

Infectious disease and ophthalmology teams were consulted. The patient was started on intravenous (IV) ampicillin/sulbactam 3 g four times a day, IV acyclovir 10 mg/kg three times a day, oral prednisone 5 mg daily, and bacitracin ophthalmic ointment. Cold compresses, gabapentin 200 mg three times a day, acetaminophen, and tramadol were given as needed for additional pain control and supportive care. Her contact isolation was maintained as the vesicular rash was in various stages with active viral lesions. Upon discharge, a 14-day course of valacyclovir 1 g three times a day and prednisone 5 mg one time a day were prescribed given the severe nature of the infection. Valacyclovir 1 g three times a day was prescribed over acyclovir 800 mg five times a day for better patient compliance. Prednisone was prescribed for the swelling and acute symptoms. The primary team also discussed the need for Shingrix vaccination once acute symptoms had subsided to prevent future outbreaks and hospitalizations.

## Discussion

Treatment of HZO requires timely administration of antiviral medication in addition to supportive care for symptoms and pain management. Topical ophthalmic antibiotics should also be considered to prevent secondary bacterial infections. In this case, intravenous antibiotics were administered because severe preseptal cellulitis was clinically evident and is what likely precipitated HZO. Artificial tears and cold compresses can provide additional anti-inflammatory relief [1,2]. Although clinical studies have shown variable efficacy, oftentimes, systemic and topical corticosteroids can be used in the setting of severe infections. Thorough ophthalmologic examination and consultation are recommended as complications, although rare, can ensue. Slit-lamp examination and fundoscopy should be performed to rule out corneal or retinal involvement [4,5].

The prognosis of HZO is case-dependent. Comorbidities and immune status also affect clinical progression and complications. In rare cases, ophthalmic pathology from HZO can lead to permanent vision loss and glaucoma. Another point of consideration is postherpetic neuralgia once HZO has resolved. Postherpetic neuralgia is a common complication with HZO and should be managed medically to reduce chronic sequelae. Clinical recognition and prompt treatment reduce the risk of HZO complications [1,2].

Routine childhood vaccinations have significantly decreased the incidence of primary VZV infections, resulting in fewer people at risk for HZ reactivation. Of the patients with HZ infection, only 10%-20% will present with HZO, and about 50% of HZO cases have true ocular disease. Although primary VZV infections have been in decline, the incidence of HZ has been increasing, which has been theorized to be a result of the growing world population of elderly [1]. Patients with a history of prior VZV infection are prone to viral reactivation as the virus enters a latent stage within the dorsal root ganglion after the initial primary infection. After primary VZV infection, the virus travels in a retrograde route on the axons of sensory nerves where it will eventually rest within the sensory nerve ganglia until provoked by periods of stress or immunosuppression [1]. During reactivation, the virus moves in an anterograde direction toward its corresponding cutaneous innervation of the skin where it will erupt as a vesicular and painful rash [4].

## Conclusions

This case displays the importance of recognizing the clinical presentation of HZO, especially in the context of underlying cellulitis. A thorough history and ocular physical examination must be conducted to determine the extent of trigeminal nerve involvement. The appropriate treatment must be administered as soon as possible or when HZO is clinically suspected because of the potential risk of permanent vision loss and postherpetic neuralgia. Prompt HZO identification in a hospital setting is especially important so that precautionary protocols may be implemented to prevent transmission to other patients and healthcare staff. Although HZO is rare, this case also highlights the importance of individuals with previous primary VZV infection receiving vaccinations for HZ. Elderly and immunocompromised individuals are especially considered high risk for HZ, and primary prevention should be stressed. Educating patients on the risks of HZ and HZO and preventative measures ensure decreased morbidity.
